# Correction: Utility of a patient similarity-based digital tool for risk communication to patients with type 2 diabetes mellitus: Perspectives from primary care physicians in ambulatory care

**DOI:** 10.1371/journal.pone.0330120

**Published:** 2025-08-11

**Authors:** Ruiheng Ong, Chirk Jenn Ng, Kalaipriya Gunasekaran, Hang Liu, Wynne Hsu, Mong Li Lee, Ngiap Chuan Tan

The following information is missing from the Funding statement: This research/project is supported by the National Research Foundation, Singapore under its AI Singapore Programme (AISG Award No: AISG-GC-2019–001-2B). The sponsors or funders did not play any role in the study design, data collection and analysis, decision to publish, or preparation of the manuscript.

The following information is missing from the Competing Interests section: I have read the journal’s policy and the authors of this manuscript have the following competing interests: WH, MLL and NCT are Principal Investigator and Co-Principal Investigators respectively of “JARVIS-DHL: Transforming Chronic Care for Diabetes, Hypertension and HyperLipidemia (DHL) with AI”. This research/project is supported by the National Research Foundation, Singapore under its AI Singapore Programme (AISG Award No: AISG-GC-2019–001-2B). This does not alter our adherence to PLOS ONE policies on sharing data and materials.
