# Supplementary material for: Utility of a patient similarity-based digital tool for risk communication to patients with type 2 diabetes mellitus: perspectives from primary care physicians in ambulatory care
Source: PLoS One. 2025 Mar 18;20(3):e0319992. doi: 10.1371/journal.pone.0319992 (PMC11918407; doi:10.1371/journal.pone.0319992)
Supplement: S1 Appendix — (PDF) [file pone.0319992.s001.pdf]

Consolidated criteria for reporting qualitative studies (COREQ): 32-item checklist

Manuscript Title: Utility of a patient similarity-based digital tool for risk communication to patients with type 2 diabetes mellitus: perspectives from primary care physicians in ambulatory care

No. Item	Guide questions/description	Section (and page #) reported
Domain 1: Research team and reflexivity		
Personal Characteristics		
1. Interviewer/facilitator	Which author/s conducted the interview or focus group?<br><br>RO conducted the interviews.	Data collection (page 5)
2. Credentials	What were the researcher’s credentials? E.g. PhD, MD<br><br>MD – KG<br>MMed – RO, NCT<br>MSc – HL<br>PhD – CJN, WH, MLL	Study team and reflexivity (page 4)
3. Occupation	What was their occupation at the time of the study?<br><br>Family Physicians – RO, CJN, NCT<br>Research Associate – KG<br>Computer scientists – HL, WH, MLL	Study team and reflexivity (page 4)
4. Gender	Was the researcher male or female?<br><br>The study team comprised both male (RO, CJN, NCT) and female (KG, HL, WH, MLL) members.	Study team and reflexivity (page 4)
5. Experience and training	What experience or training did the researcher have?<br><br>RO, KG, CJN and NCT are trained in qualitative research, while CJN and NCT are experienced primary care researchers and hold professorial appointments in Family Medicine.	Study team and reflexivity (page 4)
Relationship with participants		
6. Relationship established	Was a relationship established prior to study commencement?<br><br>Two of the eleven PCP participants had no prior relationship with RO. Two PCPs have	Participants and sampling (page 4)

	taken part in clinical workgroup activities with RO in a non-academic setting. The remaining seven PCPs knew RO only at an acquaintance level due to the relatively smaller number of Family Physicians within the institution. Most of the PCPs knew CJN and NCT as academic appointment holders in the institution's research department.	
7. Participant knowledge of the interviewer	What did the participants know about the researcher? e.g. personal goals, reasons for doing the research<br><br>RO introduced himself to each PCP participant as the Principal Investigator of the study and explained the PERDICT.AI tool and purpose of the study. Assurance was given about data confidentiality.	Participants and sampling (page 4)
8. Interviewer characteristics	What characteristics were reported about the inter viewer/facilitator? e.g. Bias, assumptions, reasons and interests in the research topic<br><br>As a practising PCP in a clinic with a high volume of patient visits, RO was cognisant of barriers that PCPs practising in similar environments would face towards adopting new technology. RO reflected on his dual role as a researcher and clinician and had ongoing discussions on these with CJN and NCT. RO actively switched his attention away from his clinician role and immersed himself into the researcher role, making conscientious efforts to maintain distance from his personal views during data collection and analysis. RO remained receptive to understanding positive and negative perspectives given by the PCP participants. PCP participants, especially those of lower seniority to RO, were encouraged during the interviews to share their honest responses and highlight issues faced during usage of PERDICT.AI that would inform the study team on areas to improve the tool. Open-ended questions were used, and leading questions were avoided. Active listening and verbal cues were used to encourage participants to articulate their views. Where needed, paraphrasing and clarification was done to	Study team and reflexivity (page 4)

	ensure accurate understanding of participants' responses. CJN was present when RO conducted the first interview, and a debriefing was done post-session.	
Domain 2: study design		
Theoretical framework		
9. Methodological orientation and Theory	What methodological orientation was stated to underpin the study? e.g. grounded theory, discourse analysis, ethnography, phenomenology, content analysis<br><br>Qualitative study design, thematic analysis<br><br>Andrew Smart's multidimensional model of clinical utility, which takes into consideration the care provider's view on appropriateness and acceptability of the clinical intervention of interest, was used.	Study design and setting (page 3), Research instruments (page 4-5), Data analysis (page 5-6)
Participant selection		
10. Sampling	How were participants selected? e.g. purposive, convenience, consecutive, snowball<br><br>Purposive sampling	Participants and sampling (page 4)
11. Method of approach	How were participants approached? e.g. face-to-face, telephone, mail, email<br><br>Email followed by face-to-face recruitment	Participants and sampling (page 4)
12. Sample size	How many participants were in the study?<br><br>Eleven	Participants and sampling (page 4), Participant characteristics (page 6)
13. Non-participation	How many people refused to participate or dropped out? Reasons?<br><br>Five PCPs declined to participate, citing unavailability of time.<br><br>All eleven participants completed the study.	Data collection (page 5)
Setting		
14. Setting of data collection	Where was the data collected? e.g. home, clinic, workplace<br><br>Study site clinic	Data collection (page 5)
15. Presence of non-	Was anyone else present besides the	Data collection (page

participants	participants and researchers?<br><br>No non-participants were present.	5)
16. Description of sample	What are the important characteristics of the sample? e.g. demographic data, date<br><br>These are described in Table 1.	Participant characteristics (page 6)
Data collection		
17. Interview guide	Were questions, prompts, guides provided by the authors? Was it pilot tested?<br><br>An in-depth (semi-structured) interview topic guide was used.	Research instruments (page 4-5)
18. Repeat interviews	Were repeat interviews carried out? If yes, how many?<br><br>There were no repeat interviews.	Data collection (page 5)
19. Audio/visual recording	Did the research use audio or visual recording to collect the data?<br><br>Audio recording	Data collection (page 5)
20. Field notes	Were field notes made during and/or after the interview or focus group?<br><br>Field notes were taken during the session and at the end of the interview.	Data collection (page 5)
21. Duration	What was the duration of the interviews or focus group?<br><br>30-60 minutes	Data collection (page 5)
22. Data saturation	Was data saturation discussed?<br><br>Thematic saturation was reached.	Data analysis (page 5-6)
23. Transcripts returned	Were transcripts returned to participants for comment and/or correction?<br><br>No – All audio recordings were transcribed verbatim and checked for completeness and accuracy.	Data analysis (page 5-6)
Domain 3: analysis and findings		
Data analysis		
24. Number of data coders	How many data coders coded the data?<br><br>Two	Data analysis (page 5-6)
25. Description of the coding tree	Did authors provide a description of the coding tree?	Data analysis (page 5-6)

	Yes	
26. Derivation of themes	Were themes identified in advance or derived from the data?<br><br>Themes were derived from the data.	Data analysis (page 5-6)
27. Software	What software, if applicable, was used to manage the data?<br><br>NVivo	Data analysis (page 5-6)
28. Participant checking	Did participants provide feedback on the findings?<br><br>No – This is discussed as a study limitation.	Discussion (page 9-11)
Reporting		
29. Quotations presented	Were participant quotations presented to illustrate the themes/findings? Was each quotation identified? e.g. participant number	Principal findings (page 6-9)
30. Data and findings consistent	Was there consistency between the data presented and the findings?	Results (page 6-9), Discussion (page 9-10)
31. Clarity of major themes	Were major themes clearly presented in the findings?	Principal findings (page 7-9)
32. Clarity of minor themes	Is there a description of diverse cases or discussion of minor themes?<br><br>No minor themes emerged from data.	N/A
