# Supplementary material for: Utility of a patient similarity-based digital tool for risk communication to patients with type 2 diabetes mellitus: perspectives from primary care physicians in ambulatory care
Source: PLoS One. 2025 Mar 18;20(3):e0319992. doi: 10.1371/journal.pone.0319992 (PMC11918407; doi:10.1371/journal.pone.0319992)
Supplement: S2 Appendix — This was used during in-depth interviews to explore participants’ views on the appropriateness and acceptability of the PERDICT.AI digital tool and to identify the challenges encountered. (PDF) [file pone.0319992.s002.pdf]

Topic guide

Ask about overall experience of PERDICT.AI

Ask about usefulness of individual modules in PERDICT.AI
- HBA1c rank
- Complication prevalence among patient cohort, case narratives
- Medication recommender module
- Care plan section
- Overall

Probe on specific direct observations

Ask about appropriateness of ranking HBA1c against a patient cohort

Specify which variables are most important to illustrate the idea of patient similarity

Relevant patient subgroups and clinic setting to use PERDICT.AI

Ask about mandatory usage vs allowing flexibility for selective usage

Acceptability of PERDICT.AI in routine practice

Foreseeable barriers to adoption

Suggestions for improvement

Knowledge and/or training gaps limiting use of PERDICT.AI

Check if participant has anything else he/she would like to share before closing the interview
