# Supplementary material for: Utility of a patient similarity-based digital tool for risk communication to patients with type 2 diabetes mellitus: perspectives from primary care physicians in ambulatory care
Source: PLoS One. 2025 Mar 18;20(3):e0319992. doi: 10.1371/journal.pone.0319992 (PMC11918407; doi:10.1371/journal.pone.0319992)
Supplement: S3 Appendix — This is a detailed description of modules in the PERDICT.AI digital tool. (PDF) [file pone.0319992.s003.pdf]

PERDICT.AI digital tool

Module	Description	Post-study Adaptations and Rationale
1: Individual-level HBA1c control and Peer-level HBA1c comparison ("Peer comparison")	Patient's HBA1c is placed into a colour-coded category of control (individual-level HBA1c control) and ranked amongst a cohort[a] of patients with diabetes (peer-level HBA1c comparison) at the primary care institute	Individual-level HBA1c control and peer-level HBA1c comparison discussed separately using two independent diagrams, in order to minimise confusion<br><br>The similar patient subgroup replaces diabetes cohort for peer-level HBA1c ranking, in order to allow loss-framing and gain-framing when discussing complications among the similar patient subgroup
2A: Complications among the similar patient subgroup	Patient is shown the % prevalence of diabetes complications among a subgroup of patients similar to him/her (i.e. similar patient subgroup), identified using a K-nearest neighbour similarity algorithm	The similar patient subgroup is further split into two subgroups based on HBA1c and compare the complication prevalence between the two subgroups (i.e. subgroup analysis), in order to allow loss-framing and gain-framing<br><br>Relevant diabetes complication(s) – especially endpoints – can be explained to patient, in order to establish common shared understandings of health outcomes
2B: Case narrative of similar patients	Patient is shown an example of suboptimal control (i.e. suboptimal diabetes control and more complications developed) and good control (i.e. good diabetes control and fewer complications developed) from the similar patient subgroup	Fewer HBA1c data points (over longer time intervals) displayed and graphs of both case examples superimposed, in order to reduce information fatigue while retaining emphasis on the contrasting HBA1c trajectories and complication outcomes between case examples
3: Medication recommender	Diabetes medications are recommended according to prescription frequency among the similar patient subgroup from the similarity algorithm<br><br>Information is provided on the expected HBA1c effect, cost and side effects of each medication; can be compared with alternative medications	Modification to a rule-based medication recommender algorithm in accordance to local clinical practice guidelines, in order to factor in step-wise medication adjustment and patient factors (e.g. co-morbidities)
4: Care plan	Doctor and patient co-create a personalised care plan with action measures (diet, exercise, medications and monitoring plans) and setting of targets (HBA1c, weight)	Generic advice replaced with specific actionable tasks relevant to patient, in order for a more patient-centred care plan

[a]Based on de-identified data from electronic medical records over a 10-year period (1 April 2009 to 31 March 2019)
