# Supplementary figures and images for: Utility of a patient similarity-based digital tool for risk communication to patients with type 2 diabetes mellitus: perspectives from primary care physicians in ambulatory care
Source: PLoS One. 2025 Mar 18;20(3):e0319992. doi: 10.1371/journal.pone.0319992 (PMC11918407; doi:10.1371/journal.pone.0319992)

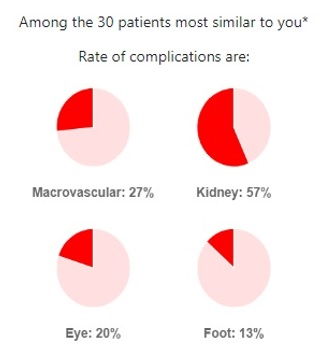

Supplement: S4 Appendix — (TIF) [file pone.0319992.s004.tif]

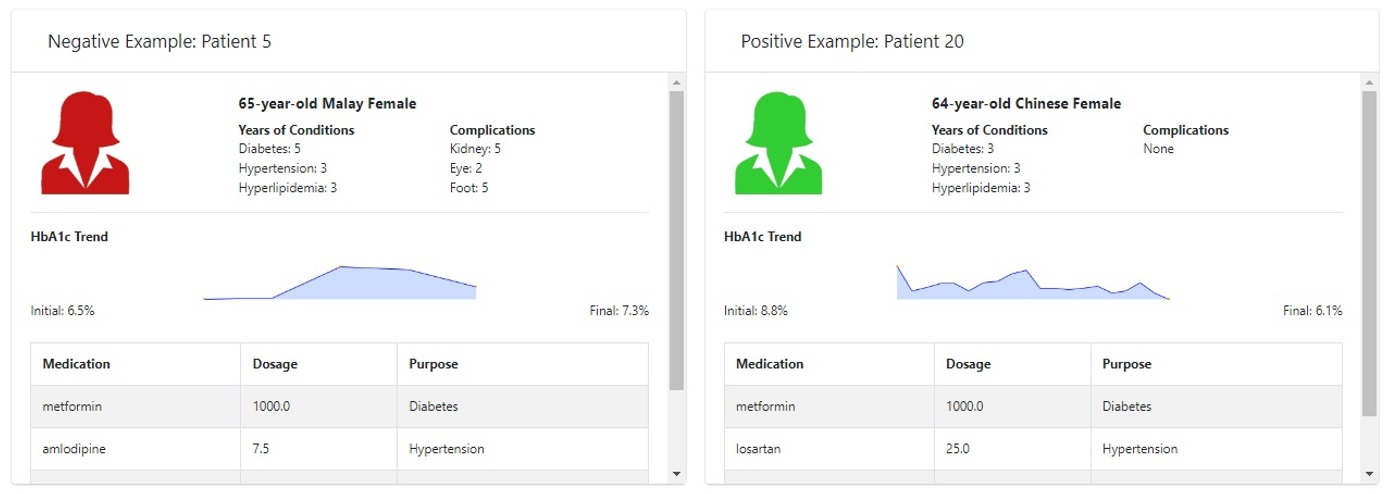

Supplement: S5 Appendix — (TIF) [file pone.0319992.s005.tif]
