# Supplementary material for: Utility of a patient similarity-based digital tool for risk communication to patients with type 2 diabetes mellitus: perspectives from primary care physicians in ambulatory care
Source: PLoS One. 2025 Mar 18;20(3):e0319992. doi: 10.1371/journal.pone.0319992 (PMC11918407; doi:10.1371/journal.pone.0319992)
Supplement: S6 Appendix — (PDF) [file pone.0319992.s006.pdf]

**Hypothetical patient case scenarios for use in the PERDICT.AI digital tool**

| Scenario | Medical problems (Year diagnosed) | Latest clinical parameters and lab results | Current medications |
|---|---|---|---|
| 50-year-old Chinese Male | Type 2 diabetes (2020)<br>Hyperlipidaemia (2016) | Height 170 cm Weight 68 kg<br>BP[1] 125/75 mmHg<br>HBA1c[2] 7.5% (4 months ago: 7.1%)<br>LDL[3] 2.2 mmol/L HDL[4] 1.2 mmol/L<br>TG[5] 1.6 mmol/L | Metformin 250mg BD[6]<br>Simvastatin 10mg ON[7] |
| 55-year-old Indian Female | Type 2 diabetes (2018)<br>Hypertension (2008)<br>Hyperlipidaemia (2006) | Height 155 cm Weight 52 kg<br>BP[1] 110/60 mmHg<br>HBA1c[2] 8.0% (4 months ago: 7.8%)<br>LDL[3] 2.4 mmol/L HDL[4] 1.0 mmol/L<br>TG[5] 1.8 mmol/L | Metformin 500mg BD[6]<br>Glipizide 5mg BD[6] pre-meal<br>Losartan 50mg OM[8]<br>Atorvastatin 10mg ON[7] |
| 60-year-old Malay Male | Type 2 diabetes (2014)<br>Hypertension (2006)<br>Hyperlipidaemia (2000) | Height 168 cm Weight 72 kg<br>BP[1] 120/70 mmHg<br>HBA1c[2] 8.5% (4 months ago: 8.8%)<br>LDL[3] 2.3 mmol/L HDL[4] 0.9 mmol/L<br>TG[5] 2.0 mmol/L | Metformin 1g BD[6]<br>Glipizide 15mg BD[6] pre-meal<br>Dapagliflozin 10mg OM[8]<br>Enalapril 20mg BD[6]<br>Nifedipine LA 60mg OM[8]<br>Atorvastatin 10mg ON[7] |

[1]Blood pressure

[2]Glycated haemoglobin; Haemoglobin A1c

[3]Low-density lipoprotein

[4]High-density lipoprotein

[5]Triglyceride

[6]Twice daily

[7]Once every night

[8]Once every morning
